# Association of Diabetes Mellitus With a Shared Hyperinflammatory Immune Response in Patients With Melioidosis and Patients With Tuberculosis: An Observational Case-Control Study

**DOI:** 10.1093/ofid/ofag286

**Published:** 2026-06-17

**Authors:** Patpong Rongkard, Barbara Kronsteiner, Clare Eckold, Parinya Chamnan, Suchintana Chumseng, Mohammad Ali, Jennifer Hill, Priyanka Abraham, Emanuele Marchi, Direk Limmathurotsakul, Narisara Chantratita, T Eoin West, Sina A Gharib, Jacqueline M Cliff, Nicholas P J Day, Paul Klenerman, Susanna J Dunachie

**Affiliations:** NDM Centre for Global Health Research, Nuffield Department of Clinical Medicine, University of Oxford, Oxford, United Kingdom; Mahidol-Oxford Tropical Medicine Research Unit, Mahidol University, Bangkok, Thailand; NDM Centre for Global Health Research, Nuffield Department of Clinical Medicine, University of Oxford, Oxford, United Kingdom; Mahidol-Oxford Tropical Medicine Research Unit, Mahidol University, Bangkok, Thailand; Tuberculosis Centre and Department of Infection and Biology, London School of Hygiene and Tropical Medicine, London, United Kingdom; Cardiometabolic Research Group, Department of Social Medicine, Sunpasitthiprasong Hospital, Ubon Ratchathani, Thailand; Mahidol-Oxford Tropical Medicine Research Unit, Mahidol University, Bangkok, Thailand; NDM Centre for Global Health Research, Nuffield Department of Clinical Medicine, University of Oxford, Oxford, United Kingdom; Mahidol-Oxford Tropical Medicine Research Unit, Mahidol University, Bangkok, Thailand; Department of Biochemistry and Molecular Biology, Bangabandhu Sheikh Mujib Medical University, Dhaka, Bangladesh; NDM Centre for Global Health Research, Nuffield Department of Clinical Medicine, University of Oxford, Oxford, United Kingdom; Mahidol-Oxford Tropical Medicine Research Unit, Mahidol University, Bangkok, Thailand; NDM Centre for Global Health Research, Nuffield Department of Clinical Medicine, University of Oxford, Oxford, United Kingdom; Mahidol-Oxford Tropical Medicine Research Unit, Mahidol University, Bangkok, Thailand; Peter Medawar Building for Pathogen Research, Nuffield Department of Clinical Medicine, University of Oxford, Oxford, United Kingdom; Mahidol-Oxford Tropical Medicine Research Unit, Mahidol University, Bangkok, Thailand; Department of Microbiology, Mahidol University, Bangkok, Thailand; Division of Pulmonary, Critical Care and Sleep Medicine, University of Washington, Seattle, Washington, USA; Department of Global Health, University of Washington, Seattle, Washington, USA; Division of Pulmonary, Critical Care and Sleep Medicine, University of Washington, Seattle, Washington, USA; Tuberculosis Centre and Department of Infection and Biology, London School of Hygiene and Tropical Medicine, London, United Kingdom; NDM Centre for Global Health Research, Nuffield Department of Clinical Medicine, University of Oxford, Oxford, United Kingdom; Mahidol-Oxford Tropical Medicine Research Unit, Mahidol University, Bangkok, Thailand; Peter Medawar Building for Pathogen Research, Nuffield Department of Clinical Medicine, University of Oxford, Oxford, United Kingdom; Translational Gastroenterology and Liver Unit, Nuffield Department of Clinical Medicine, University of Oxford, Oxford, United Kingdom; NIHR Oxford Biomedical Research Centre, Oxford University Hospitals NHS Foundation Trust, Oxford, United Kingdom; NDM Centre for Global Health Research, Nuffield Department of Clinical Medicine, University of Oxford, Oxford, United Kingdom; Mahidol-Oxford Tropical Medicine Research Unit, Mahidol University, Bangkok, Thailand; Peter Medawar Building for Pathogen Research, Nuffield Department of Clinical Medicine, University of Oxford, Oxford, United Kingdom; NIHR Oxford Biomedical Research Centre, Oxford University Hospitals NHS Foundation Trust, Oxford, United Kingdom

**Keywords:** diabetes mellitus, host-pathogen response, melioidosis, transcriptome, tuberculosis

## Abstract

**Background:**

Melioidosis is a serious infection caused by the bacterium *Burkholderia pseudomallei* with a case fatality rate of up to 40% in Northeast Thailand. Diabetes mellitus (DM) increases the risk of developing melioidosis by 12-fold. A similar, but less marked relationship with DM is seen in patients with tuberculosis, with a 3-fold increased risk of developing tuberculosis in people with DM. However, the mechanisms underlying the impact of DM on infection are not fully understood.

**Methods:**

Eighty-one patients with acute melioidosis from Northeast Thailand and 151 patients with tuberculosis from South Africa, Indonesia, Romania, and Peru, along with uninfected control cohorts, were studied by whole-blood RNA sequencing. Both supervised and unsupervised data analysis approaches, were performed including differential gene expression, pathway, and weighted gene coexpression network analyses.

**Results:**

DM status was associated with a hyperinflammatory response to both melioidosis and tuberculosis, with increased neutrophil and platelet degranulation and exaggerated activation of coagulation and scavenger activation pathways, along with decreased phosphoinositide 3-kinase protein kinase B signaling. In melioidosis, changes with DM were subtle but also included increased tumor necrosis factor signaling via nuclear factor κB and enhancement of endoplasmic reticulum stress and unfolded protein responses. DM-related changes were more distinct in tuberculosis, with marked reduction of interferon signaling responses.

**Conclusions:**

DM is associated with enhanced nonspecific inflammatory responses in both melioidosis and tuberculosis and an impaired interferon-mediated response to tuberculosis, with implications for future host-directed therapies.

## Teaser

Diabetes mellitus is associated with hyperinflammatory responses to both melioidosis and tuberculosis, with increased neutrophil and platelet degranulation and exaggerated activation of coagulation and scavenger activation pathways. The impact is more subtle in melioidosis but pronounced in tuberculosis with stunted interferon responses.

Melioidosis is a deadly infectious disease caused by infection with *Burkholderia pseudomallei* (Bp), a gram-negative bacillus with estimates of 165 000 cases and 89 000 deaths globally each year [1]. The case fatality rate for people admitted to the hospital with melioidosis is up to 40% in Northeast Thailand and 14% in Australia [2, 3]. Risk factors include chronic renal disease, excessive alcohol consumption, older age and diabetes mellitus (DM), which carries a 12-fold increased risk [2, 3].

*Mycobacterium tuberculosis* (Mtb) causes tuberculosis and is the leading cause of death from a single pathogen worldwide (tuberculosis) [4]. In 2023, almost 11 million people were infected with Mtb, with an estimated 1.09 million deaths [4]. Risk factors for the development of tuberculosis disease include human immunodeficiency virus (HIV) infection, undernourishment, alcohol abuse, smoking, and DM [5]. DM is associated with up to a 3-fold increased risk of developing tuberculosis [6] and contributed to approximately 1 million tuberculosis cases worldwide in 2013 [7]. It is well established that individuals with DM face an increased risk of tuberculosis, particularly in regions with high tuberculosis prevalence [8].

Both melioidosis and tuberculosis are caused by intracellular bacteria, typically present with lung disease, and both feature granuloma formation [9]. Type I immune responses are important for controlling both infections [10, 11]. The interferon (IFN) γ response in melioidosis shifts from primary natural killer (NK) cell and CD8 T-cell sources during the acute phase to a CD4/CD8 T-cell–driven enhancement in recovered or endemic individuals [12, 13]. In contrast, protective immunity against Mtb critically relies on CD4 T-cell–derived IFN-γ to enable effective CD8 T-cell function [14]. These differences in the dominant cellular sources and dependencies of the protective IFN-γ response suggest contrasting host immune dynamics, likely driven by both bacterial pathogenicity [11, 15] and preexisting host factors and immune status [16, 17].

In people with DM, multiple host immune response pathways are compromised and dysregulated [18], and macrophages, monocytes, and neutrophils show compromised phagocytosis and antimicrobial capacity [19–21]. Compared with patients with tuberculosis but without DM, those with both tuberculosis and DM also show systemic elevation of proinflammatory cytokines [22]. However, IFN-γ cellular immune responses within the lungs were reduced and delayed in a mouse DM model during Mtb challenge [23]. Overall, common pathways underlying the impact of DM on the immune response to Bp and Mtb have not been well characterized. We hypothesized that the increased risk of Bp and Mtb infection among patients with DM is underlined by common transcriptomic profiles during infection. In the current study, we examined the relationship between whole-blood transcriptomic profiles associated with DM during melioidosis and tuberculosis.

## METHODS

### Study Design and Ethical Approval

For the melioidosis cohort, we conducted a prospective observational study (MICRO1501) between 2015 and 2017 at Sunpasitthiprasong Hospital, Ubon Ratchathani, Thailand. The study protocol was approved by the ethics committees of the Faculty of Tropical Medicine, Mahidol University (TMEC 12014) and Sunpasitthiprasong Hospital, Ubon Ratchathani (017/2559) and the Oxford Tropical Research Ethics Committee (OXTREC35-15). We recruited adults aged ≥18 years into the melioidosis cohort of the study as soon as feasible after hospitalization with melioidosis, defined as culture of Bp from any clinical specimen. We also recruited healthy household contacts of melioidosis case patients enrolled in the study as endemic control participants to minimize demographic bias between case patients and controls, alongside people with DM recruited from the DM outpatient clinic at Sunpasitthiprasong Hospital. We measured the hemoglobin A_1c_ (HbA_1c_) level for all participants and defined DM status for the melioidosis cohort as having a preexisting diagnosis of DM and/or an HbA_1c_ level ≥6.5%, according to World Health Organization criteria [24].

For the tuberculosis cohort, we used whole-blood bulk RNA sequencing data from a previously published study [25, 26]. This study included 239 adult participants, aged ≥18 years with newly diagnosed, bacteriologically confirmed pulmonary tuberculosis and with or without DM, along with uninfected patients with DM and healthy donors. Recruitment occurred between 2013 and 2016 across 4 study sites: South Africa, Indonesia, Romania, and Peru [26]. The patients with tuberculosis were further classified as (1) “tuberculosis only” (tuberculosis without DM; HbA_1c_ <5.7%), (2) “tuberculosis-IH” (tuberculosis with intermediate hyperglycemia [IH]; HbA_1c_ between 5.7% and <6.5%), or (3) “tuberculosis-DM” (tuberculosis with DM; (HbA_1c_ ≥6.5%). The study size was selected based on samples available and in line with successful studies yielding findings of interest in the peer-reviewed literature. All studies were conducted according to Good Clinical Practice, and all participants gave written informed consent, including for export and storage of their blood samples.

### Sample Collection

For the melioidosis cohort, we collected 3-mL whole-blood samples into Tempus blood RNA tubes (Applied Biosystems) from patients with culture-confirmed melioidosis (melioidosis cohort; n = 81) along with control cohorts, outpatients with DM (n = 15), and household contacts of the patients with melioidosis (n = 14). The Tempus blood RNA tubes were stored at −80°C until further processing.

### RNA Sequencing and Data Acquisition

For the melioidosis cohort, we isolated total RNA from whole-blood samples collected in Tempus blood RNA tubes using the Tempus Spin RNA Isolation (Applied Biosystems; Thermo Fisher Scientific). We performed library preparation and RNA sequencing at the Oxford Genomics Centre, Wellcome Centre for Human Genetics, with Ribo-Zero library preparation and the globin depletion workflow, and we sequenced the library for 75–base pair paired-end sequences with 50 million reads using a HiSeq4000 sequencer. For the tuberculosis cohort, Eckold et al [25] generated the expression data and provided the raw sequencing data (FASTQ files). Briefly, whole blood was collected into PAXgene Blood RNA Tubes (PreAnalytiX; Qiagen) and sequenced from the polyA tail library preparation approach. For this study we used the same upstream data analysis pipeline—including quality check, mapping, and read counting—for both melioidosis and tuberculosis cohorts (Supplementary materials).

### Statistical Analysis

**Figure 1:**
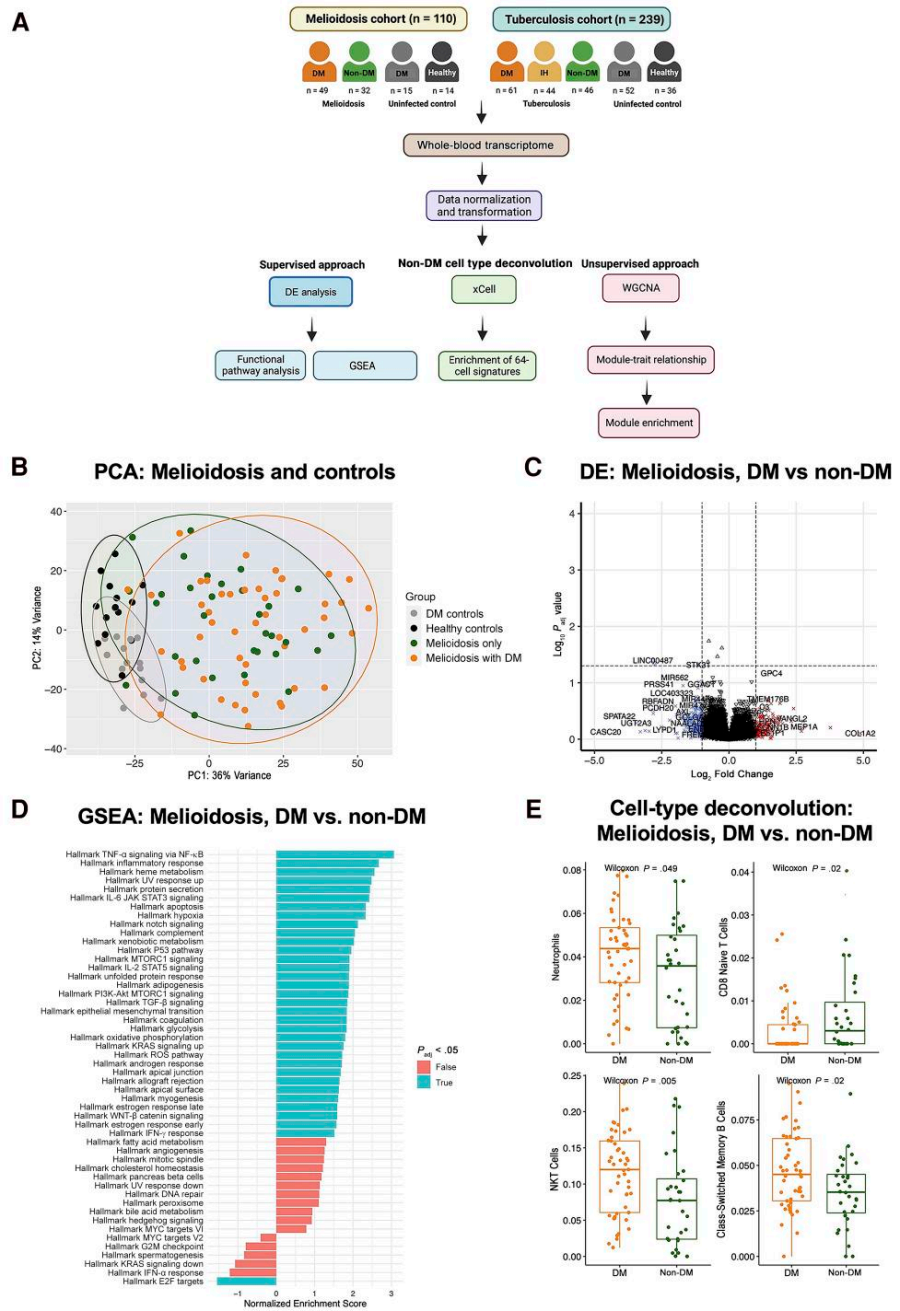
Data analysis pipeline and whole-blood transcriptomic profiling in patients with acute melioidosis and diabetes mellitus (DM). *A*, Core data analysis approaches for both melioidosis and tuberculosis study cohorts consist of a supervised approach, cell type deconvolution, and an unsupervised approach. Abbreviations: DE, differential gene expression; GSEA, gene set enrichment analysis; IH, intermediate hyperglycemia; WGCNA, weighted gene coexpression network analysis. *B*, Principal component (PC) analysis (PCA) of the top 1000 most variable genes among patients with melioidosis and control cohorts. The melioidosis cohort is divided into patients with DM (*orange dots*; n = 49), patients without DM (melioidosis only) (*green dots*; n = 32), uninfected outpatients with DM (DM controls) (*gray dots*; n = 15), and uninfected healthy donors (healthy controls) (*gray dots*; n = 14). *C*, Volcano plot of differentially expressed genes (DEGs) between melioidosis with or without DM. DEGs were based on absolute (log_2_ fold change) ≥1 (x-axis) and adjusted *P* (*P*_adj_) < .05 (y-axis) (*dotted lines*). *D*, Gene set enrichment analysis between patients with melioidosis with or without DM based on Hallmark gene sets. Normalized enrichment scores are displayed where significant pathways were labeled in turquoise (false discovery rate, <0.05). Abbreviations: G2M checkpoint, progression through the G2/M checkpoint cell division cycle; IFN, interferon; IL-2 and IL-6, interleukin 2 and 6; JAK, janus kinase; KRAS, Kirsten rat sarcoma virus oncogene homologue; NF-κB, nuclear factor κB; MTOR, mechanistic target of rapamycin; MTORC1, mechanistic target of rapamycin complex 1; MYC, myelocytoma; PI3K-Akt, phosphoinositide 3-kinase protein kinase B; ROS, reactive oxygen species; STAT, signal transducer and activator of transcription; TGF, transforming growth factor; TNF, tumor necrosis factor, WNT, wingless and Int-1. *E*, Significant immune cell-subset enrichment between patients with melioidosis with or without DM (created with BioRender.com). The statistical analysis was performed using Mann-Whitney test, and the corresponding *P* value was displayed on each plot along with median and interquartile range boxes. Abbreviation: NKT, natural killer T. Created in BioRender. Dunachie, S. (2026) https://BioRender.com/aswsb1i.

Expression data (transcripts) were annotated to gene symbols. Genes with no counts or low expression were removed. An overview of the data analysis pipeline is provided in Figure 1*A*. Briefly, differential gene expression (DE) analysis was carried out using a negative binomial generalized linear model implemented in the DESeq2 R package (version 1.34.0), adjusted for age and sex covariates [27]. Following DE analysis, functional pathway analysis was performed, using the clusterProfiler R package (version 4.6.2) [28]. Gene set enrichment analysis (GSEA) based on the MSigDB gene sets was performed using the fgsea R package (version 1.18.0) [29]. To identify coexpressed gene modules associated with DM and corresponding hub genes during melioidosis and tuberculosis, we performed weighted gene coexpression network analysis (WGCNA) [30]. Subsequently, coexpressed modules that displayed significant relationship to clinical variables were subjected to pathway enrichment analysis. Finally, to identify immune cell populations associated with DM, cell type abundances were estimated from bulk RNA sequencing data using xCell software (version 1.1.0) [31].

## RESULTS

### Profound Changes in the Whole-Blood Transcriptome Caused by Melioidosis

The melioidosis cohort consists of 81 patients with culture-proven melioidosis, in which majority of the patients (n = 49 [61%]) had DM by the time of enrollment (Table 1), alongside 29 healthy controls with or without DM (Figure 1*A*). We sampled the patients a median of 5 days (interquartile range, 4–6 days) after hospital admission. The case fatality rate was 35%, with no difference between DM and non-DM cohorts. To determine whether melioidosis causes changes in gene expression, we performed principal component analysis on the 1000 most variable genes in melioidosis and control cohorts. Principal component analysis shows a clear separation between patients with melioidosis and control groups (Figure 1*B*). There was also some separation between healthy controls and patients with DM. However, no clear separation was observed by DM status among the patients with melioidosis.

**Table 1.** Characteristics of Melioidosis and Control Cohorts in Whole-Blood Transcriptomic Study

Characteristics	Melioidosis With DM^a^ (n = 49)	Melioidosis Only (n = 32)	Healthy Controls (n = 14)	DM Controls^a^ (n = 15)	*P* Value
Female sex, no. (%)	20 (41)	7 (22)	6 (43)	8 (53)	.07^b^
Age, median (IQR), y	55 (49–63)	65 (52–73)	47 (44–54)	49 (44–57)	.01^c^
Nonsurvivors, no. (%)^d^	23 (47)	13 (41)	NA	NA	.58^b^

Abbreviations: DM, diabetes mellitus; IQR, interquartile range; NA, not applicable.

^a^DM was defined as a previous DM diagnosis or a hemoglobin A_1c_ level ≥6.5% on recruitment into the study.

^b^ *P* values based on χ^2^ test comparing patients with melioidosis with or without DM.

^c^ *P* value based on Mann-Whitney test comparing patients with melioidosis with or without DM.

^d^Case fatalities based on the 28-day mortality rate.

Next, we used DE to compare gene expression, and we identified 94 up-regulated and 2 down-regulated genes in patients with DM compared with healthy controls (absolute log_2_ fold change ≥1; adjusted *P* < .05) (Supplementary Figure 1*A*). Thus, healthy controls were used as the baseline for subsequent analyses unless stated otherwise. DE analysis identified large-scale changes in gene expression between patients with melioidosis and healthy donors with 1727 up-regulated and 799 down-regulated genes (Supplementary Figure 1*B*). Pathways involved in inflammatory immune responses, such as regulation of the complement cascade, and neutrophil and platelet degranulation, were highly up-regulated in patients with melioidosis compared with healthy control (Supplementary Figure 1*C*).

### Increased Inflammatory Immune and Cellular Stress Responses in Patients With Melioidosis and DM

DE analysis did not reveal substantial differences between patients with melioidosis with or without DM (Figure 1*C*). We next performed genome-wide functional class scoring using preranked GSEA following DE analysis. GSEA by Hallmark gene sets in patients with melioidosis and DM revealed enrichment of pathways involved in inflammation and cellular stress responses, such as tumor necrosis factor (TNF) signaling via nuclear factor κB (NF-κB), protein secretion, and heme metabolism, compared with findings in patients with melioidosis without DM (Figure 1*D*). Cell-type deconvolution analysis demonstrated that patients with melioidosis and DM, compared with those without DM, have an increased number of natural killer T (intermediate hyperglycemia) cells, class-switched memory B cells, and neutrophils, but fewer naive CD8 T cells (Figure 1*E*). We cross-validated selected differentially expressed inflammatory genes identified as elevated in melioidosis with DM by measuring serum protein levels (Supplementary Table 1). We observed a nonsignificant trend toward elevated inflammatory markers (interleukin 10, interleukin 15, placental growth factor, and soluble intercellular adhesion molecule 1) in patients with melioidosis and DM, compared with those without DM (Supplementary Figure 2).

### Association of DM During Melioidosis With Coexpressed Gene Modules Enriched for Inflammatory Immune Responses and Cellular Stress Responses

**Figure 2.**
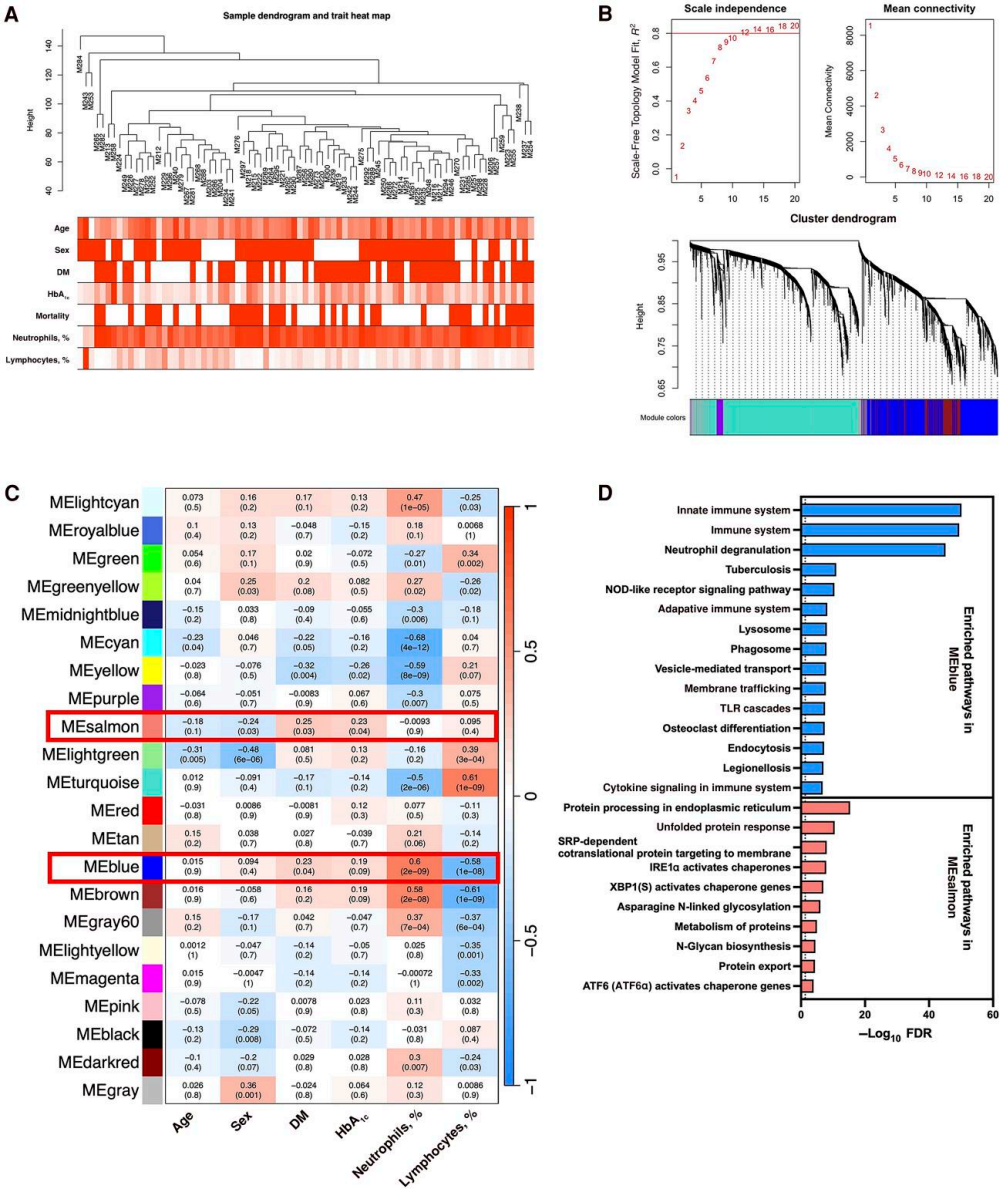
Weighted gene coexpression network analysis was performed in 81 patients with melioidosis. *A*, Sample dendrogram with corresponding clinical data. ″Height″ on y-axis represents Euclidean Distance. *B*, Selection of soft-thresholding power (ß) in Weighted Gene Co-expression Network Analysis. Analysis of scale-free topology fit index (left) and mean connectivity (right) across a range of candidate soft-thresholding powers. The x-axis represents the soft-thresholding power (β), an integer exponent applied to the absolute Pearson correlation between gene pairs. *C*, Module-trait relationship heatmap in melioidosis patients with and without diabetes. Each row represents a co-expressed gene module and each column a clinical or demographic trait. Each cell displays the Pearson correlation coefficient (r) and corresponding p-value (in parentheses). Modules with p < 0.05 were considered significantly associated with a trait. *D*, Pathway enrichment analysis within modules with significant correlation (ME blue and ME salmon) with diabetes mellitus (DM) status based on Reactome and kyoto encyclopedia of genes and genomes (KEGG) gene sets. Enriched pathways are displayed as −log_10_ false discovery rate (FDR) values, and the dotted line defines FDR <0.05. Clinical traits include age, sex, DM status, hemoglobin A_1c_ [HbA_1c_] level, and the percentages of neutrophils and lymphocytes. Abbreviations: ATF6α, activating transcription factor 6 alpha; IRE1α, inositol-requiring enzyme 1 alpha; NOD-like receptor, nucleotide-binding oligomerization domain-like receptor; SRP, signal recognition particle; XBP1(S), X-box binding protein 1, spliced form.

To complement the results obtained from DE and pathway analyses in an unsupervised manner, we performed WGCNA. A sample dendrogram was constructed to identify potential outliers (Figure 2*A*), and 22 coexpressed gene modules (module eigengenes [MEs]) were identified (each identified by a color) (Figure 2*B*). Module-trait relationship analysis identified significant correlations between DM status clinical trait and the ME salmon (Pearson ρ = 0.25; *P* = .03) and ME blue (Pearson ρ = 0.23; *P* = .04) modules (Figure 2*C*). Pathway analysis identified enrichment of pathways involved in proinflammatory immune responses such as the innate immune system, neutrophil degranulation, and the nucleotide-binding oligomerization domain-like receptor (NLR) signaling pathway within the ME blue module. Furthermore, the ME salmon module—which was associated with DM status and HbA_1c_ level—identified enriched pathways involved in the regulation of endoplasmic reticulum and the response to unfolded protein (Figure 2*D*).

### Effects of Hyperglycemia and DM on Inflammatory Immune Responses in Both Patients With Melioidosis and Patients With Tuberculosis

**Figure 3.**
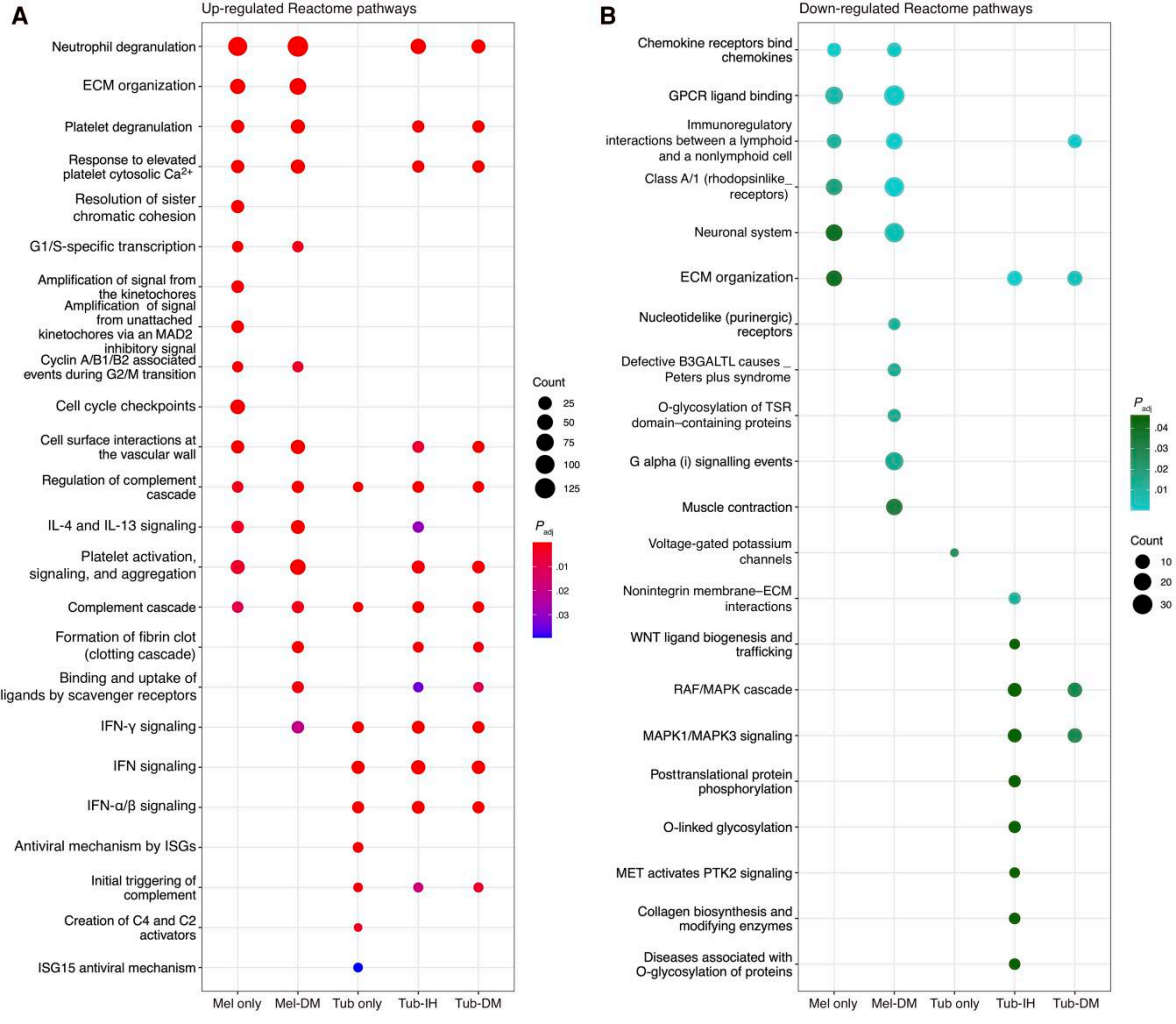
Functional pathway analysis based on Reactome gene sets following differential gene expression (DE) analyses in a South African tuberculosis cohort and a melioidosis cohort, in patients with or without diabetes mellitus (DM), displayed as up-regulated (*A*) and down-regulated (*B*) pathways in patients with melioidosis and patients with tuberculosis compared with their corresponding uninfected healthy control cohorts. Gradient color bars corresponds to adjusted *P* (*P*_adj_) values. The size of each term is indicated by representative counts (numbers of differentially expressed genes [DEGs]); DEGs were prefiltered based on a cutoff of absolute log_2_ fold change ≥1 and *P*_adj_ < .05. Mel-only and Mel-DM represent pathways derived from DE analysis between melioidosis without or with DM compared with healthy controls, respectively; Tub-only, Tub-IH, and Tub-DM, pathways derived from DE analysis of patients with tuberculosis only (without DM), tuberculosis with intermediate hyperglycemia, and tuberculosis with DM, compared with healthy controls, respectively. Abbreviations: B3GALTL, beta-1,3-glucosyltransferase; ECM, extracellular matrix; GPCR, G protein-coupled receptor; IFN, interferon; IL-4 and IL-13, interleukin 4 and 13; ISG, IFN-stimulated gene; MAPK, mitogen-activated protein kinase; MET, mesenchymal-epithelial transition factor; PTK2, protein tyrosine kinase 2; RAF, rapidly accelerated fibrosarcoma kinase.

To compare the impact of DM and IH on the whole-blood transcriptomic profiles of both tuberculosis and melioidosis compared with their respective healthy control cohorts (Figure 3), we first undertook novel analysis of a previously published transcriptomics study in tuberculosis [25], as shown in Table 2. Functional pathway analysis was used following DE analysis (absolute log_2_ fold change ≥1; adjusted *P* < .05) by Reactome and Kyoto Encyclopedia of Genes and Genomes (KEGG) gene sets from South African cohort (Supplementary Figures 3 and 4). Pathways involved in type I immune responses, such as IFN, IFN α/β, and IFN-β signaling, were up-regulated in all tuberculosis groups compared with healthy controls (Figure 3*A* and Supplementary Figure 5). However, antiviral mechanisms involving IFN-stimulated genes, IFN-stimulated gene 15 antiviral mechanisms, hepatitis C, measles, and creation of C4 and C2 activator pathways were up-regulated exclusively in the tuberculosis-only group.

**Table 2.** Baseline Demographics of Tuberculosis and Control Cohorts in Whole-Blood Transcriptomic Study, by Study Site^a^

Characteristics	Study Site (N = 239)	Tuberculosis-DM^b^ (n = 61)	Tuberculosis-IH^c^ (n = 44)	Tuberculosis Only (n = 46)	DM Controls (n = 52)	Healthy Controls (n = 36)	*P* Value^d^	*P* Value^e^
No. of participants	South Africa	15	19	11	33	24	.15^f^	.06^f^
	Romania	15	10	10	19	12		
	Indonesia	19	6	14	NA	NA		
	Peru	12	9	11	NA	NA		
Sex, male/ female, no.	South Africa	7/8	12/7	2/9	15/18	12/12	.03^g, h^	.13^g^
	Romania	13/2	9/1	6/4	14/5	10/2		
	Indonesia	11/8	5/1	7/7	NA	NA		
	Peru	6/6	5/4	5/6	NA	NA		
Age, median (IQR), y	South Africa	46 (40–53)	45 (33–54)	48 (34–50)	49 (42–57)	42 (37–45)	.43^f^	.02^f,h^
	Romania	47 (43–55)	49 (40–57)	43 (39–53)	55 (46–58)	46 (40–53)		
	Indonesia	52 (45–58)	51 (38–57)	47 (39–58)	NA	NA		
	Peru	51 (48–54)	52 (41–57)	55 (33–64)	NA	NA		
Distribution of ethnic groups, no. of groups (nos. within groups)	South Africa	2 (1/14)	1 (19)	1 (11)	1 (33)	2 (1/23)	NA	NA
	Romania	2 (1/14)	1 (10)	1 (10)	1 (19)	3 (1/1/10)		
	Indonesia	3 (1/1/17)	2 (1/5)	2 (5/9)	NA	NA		
	Peru	1 (12)	1 (9)	2 (1/10)	NA	NA		

Abbreviations: DM, diabetes mellitus; IH, intermediate hyperglycemia; IQR, interquartile range; NA, not applicable.

^a^Table adapted from Eckold et al [25].

^b^DM was defined as a previous DM diagnosis or a hemoglobin A_1c_ (HbA_1c_) level ≥6.5% on recruitment into the study [24].

^c^IH was defined as an HbA_1c_ level between 5.7% and <6.5% on recruitment into the study.

^d^ *P* values derived from statistical analyses comparing tuberculosis cohorts.

^e^ *P* values derived from statistical analyses comparing all cohorts.

^f^Calculated using Kruskal-Wallis test.

^g^Calculated using χ^2^ test.

^h^Significant at *P* < .05.

Similarly, GSEA based on Hallmark gene sets revealed that patients in the tuberculosis-IH group had highly enriched pathways involved in inflammatory immune responses and metabolism, such as coagulation, interleukin 6 JAK STAT3 signaling, complement, and reactive oxygen species (ROS), while IFN-α and IFN-β pathways were enriched in the tuberculosis-only compared with the tuberculosis-DM patient group (Supplementary Figure 5*C* and 5*D*). In a validation study using a Romanian cohort, we observed a similar pattern: patients in the tuberculosis-DM and tuberculosis-IH groups showed a greater magnitude of differentially expressed genes than those in the tuberculosis-only group (Supplementary Figure 6). Pathway analysis revealed a unique up-regulation of antiviral mechanisms, specifically IFN-stimulated genes, in the tuberculosis-only group. Consistently, GSEA demonstrated enrichment of both IFN-α and IFN-β response pathways in tuberculosis-only compared with tuberculosis-DM patients (Supplementary Figure 7).

We identified common pathways with up-regulation in DM compared with no DM for both tuberculosis (tuberculosis-DM and tuberculosis-IH) and melioidosis, such as clotting cascade and scavenger receptors pathway responsible for ligand binding and uptake. For inflammation and immune response pathways related to infection, only melioidosis with DM displayed up-regulation in TNF signaling, deficient type 1 immunity, and impaired intracellular killing mechanisms. Finally, the phosphoinositide 3-kinase protein kinase B (PI3K-Akt) signaling pathway was down-regulated in tuberculosis-IH, tuberculosis-DM, and melioidosis with DM (Supplementary Figure 5*A* and 5*B*).

### Reduced Proinflammatory Responses and Cellular Signaling in Melioidosis and Tuberculosis With DM Compared With Healthy Donors

To investigate the influence of DM on melioidosis and tuberculosis, we conducted DE and pathway analyses, comparing patients with DM-complicated melioidosis or tuberculosis to DM-only controls. In melioidosis-DM, a significant shift in gene expression was observed, with 245 genes up-regulated and 593 down-regulated (Supplementary Figure 8*A* and 8*B*). Pathway analysis revealed common inflammatory responses but a notable reduction in IFN-α/β signaling compared with DM controls (Figure 3*A* and Supplementary Figure 8*E* and 8*F*). Conversely, patients in the tuberculosis-DM group showed a smaller magnitude of gene expression change (with 64 genes up-regulated and 93 down-regulated) relative to DM controls (Supplementary Figure 8*C* and 8*D*). While IFN signaling remained intact, the suppression of rapidly accelerated fibrosarcoma (RAF)/mitogen-activated protein kinase (MAPK) and MAPK1/MAPK3 signaling pathways, was absent in tuberculosis-DM compared with DM controls (Figure 3*B* and Supplementary Figure 8*E* and 8*F*).

### Association of DM During Tuberculosis With Enriched Neutrophils and Inflammatory Immune Responses in Coexpressed Gene Modules

**Figure 4.**
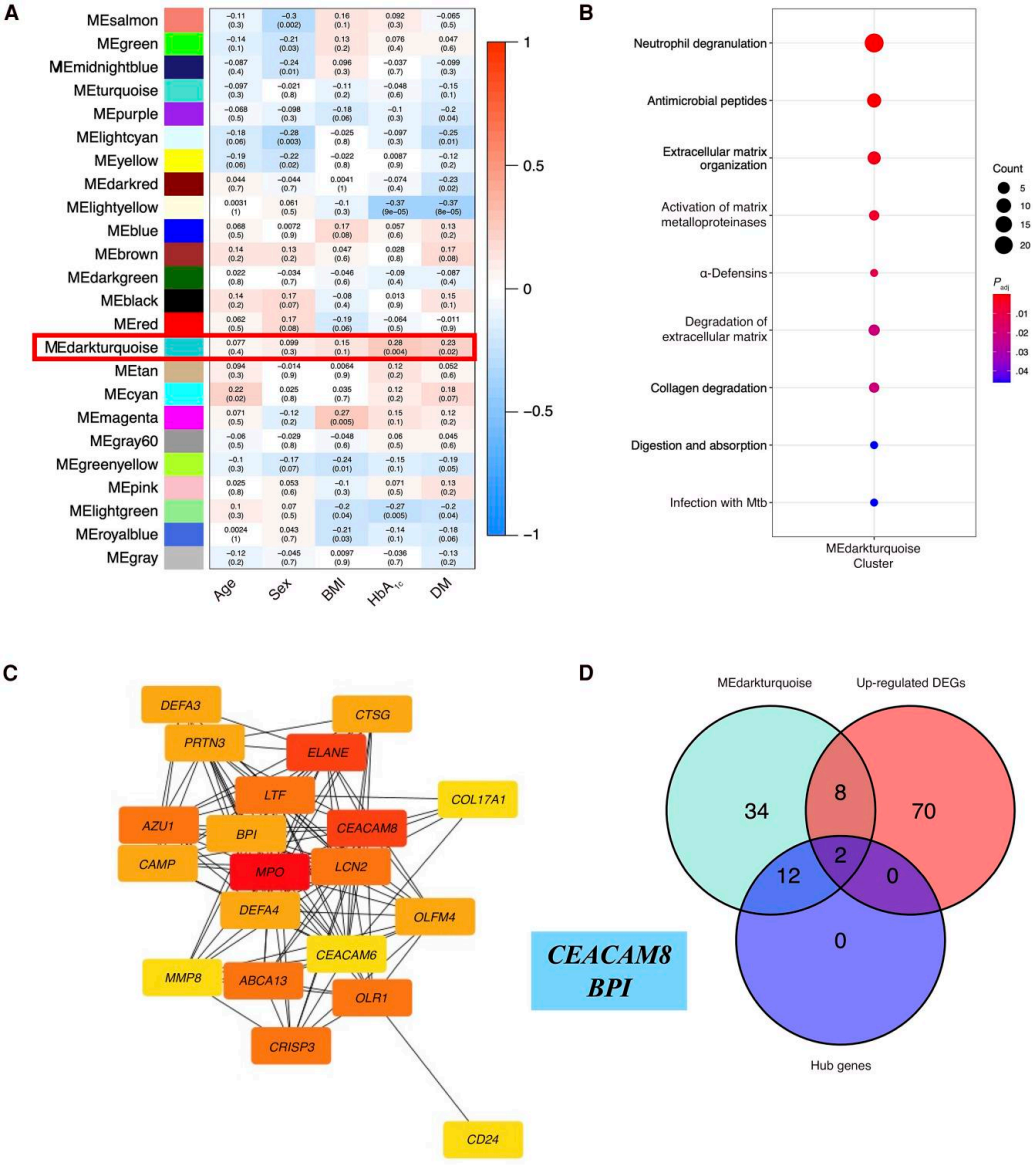
Weighted gene coexpression network analysis (WGCNA) and identification of hub genes associated with diabetes mellitus (DM) in patients with tuberculosis. *A*, Module-trait relationship heatmap in tuberculosis patients with and without diabetes. Each row represents a co-expressed gene module and each column a clinical or demographic trait. Each cell displays the Pearson correlation coefficient (r) and corresponding p-value (in parentheses). Modules with p < 0.05 were considered significantly associated with a trait. Clinical traits include age, sex, body mass index (BMI), hemoglobin A_1c_ (HbA_1c_) level, and DM status. *B*, Pathway enrichment analysis within MEs with significant correlation (ME dark turquoise) to DM status and HbA_1c_ level based on Reactome and kyoto encyclopedia of genes and genomes (KEGG) gene sets. enriched pathways are displayed as −log_10_ false discovery rate values. Abbreviations: Mtb, *Mycobacterium tuberculosis*; *P*_adj_, adjusted *P* value. *C*, Top 20 hub genes identified in the ME dark-turquoise module associated with DM status. Hub genes were identified based on the Maximal Clique Centrality (MCC) algorithm using the CytoHubba (version 0.1) plug-in in Cytoscape software (version 3.9). The MCC scores of hub genes were ranked from high (*red*) to low (*yellow*), and 6 hub genes without an MCC score were excluded. *D*, Venn diagram was constructed to show the overlap between differentially expressed genes (DEGs), ME dark-turquoise gene membership identified by weighted gene coexpression network analysis, and hub genes identified within the ME dark-turquoise module. The DEGs were identified based on genes up-regulated in patients with tuberculosis and DM, compared with tuberculosis only, using a cutoff of absolute log_2_ fold change ≥0.5 and *P*_adj_ < .05.

WGCNA comparing tuberculosis-DM and tuberculosis-only cohorts was performed to identify gene modules and hub genes that are associated with DM (Supplementary Figure 9*A*–9*D*). In all, 23 coexpressed gene modules were identified (Supplementary Figure 9*D*). Significant correlations were found between the ME dark-turquoise module and HbA_1c_ levels and DM status through module-trait relationship analysis (Figure 4*A*). Module enrichment analysis based on Reactome and KEGG gene sets within the ME dark-turquoise module (correlated with DM status and HbA_1c_ level) identified enriched pathways involved in defense against infections driven by the innate immune compartment, such as neutrophil degranulation, antimicrobial peptides, and α-defensins (Figure 4*B*). The top 20 hub genes were identified within the ME dark-turquoise module (Figure 4*C*), of which 2 genes, *CEACAM8* and *BPI*, were up-regulated in the tuberculosis-DM compared with the tuberculosis-only patient group (Figure 4*D*). Finally, neutrophils and T-helper 2 cells were significantly enriched in tuberculosis-DM, compared with tuberculosis without DM (Supplementary Figure 10).

## DISCUSSION

Despite an estimated 12-fold increased risk of melioidosis in people with DM, differences in the transcriptomic profiles between patients with melioidosis with or without DM were subtle, but we were able to characterize the influences of DM using GSEA and WGCNA. Patients with melioidosis and DM, compared with those without DM cases, showed enrichment of multiple pathways involving the inflammatory immune responses and cellular stress responses by endoplasmic reticulum. These findings of enrichment of neutrophils and inflammatory responses were also identified in the tuberculosis-DM group.

Our study demonstrates an increased neutrophil-mediated immune response that may be contributing to a disorganized immune response and detrimental inflammation. Neutrophils play a detrimental role in DM by increasing nonspecific inflammatory immune responses [32]. Hyperglycemia, a hallmark of DM, results in neutrophil dysfunction with impairments observed in chemotaxis, phagocytosis, and killing mechanisms [33]. Neutrophils under hyperglycemic conditions are highly susceptible to NETosis, contributing to inflammation and tissue damage [34].

Our analysis confirms that the transcriptome of whole blood from patients with tuberculosis is dominated by IFN signaling pathways and the complement cascade. However, patients with tuberculosis-DM and those with tuberculosis-IH show reduced expression of IFN signaling genes and pathways, compared with tuberculosis-only patients. In addition, patients in the tuberculosis-DM and tuberculosis-IH groups show up-regulated pathways involved in inflammation driven by the innate immune compartment. Patients with melioidosis have similar transcriptomic profiles, with up-regulation of pathways involved in inflammatory immune responses. IFN-γ is crucial for controlling intracellular infections like tuberculosis and melioidosis [10, 35]. In tuberculosis, IFN-γ production by CD4 T cells is essential and irreplaceable by other immune cell types. HIV, which depletes CD4 T cells, significantly increases susceptibility to Mtb infection and reactivation of latent tuberculosis [36]. However, in melioidosis, NK cells and CD8 T cells are the primary sources of IFN-γ [12, 13]. Consequently, the impact of HIV infection on melioidosis susceptibility is likely mitigated by these alternative IFN-γ sources, consistent with the lack of observed increased susceptibility [37]. Overall, these findings suggest that type I immune responses are important for controlling both tuberculosis and melioidosis, but the mechanisms by which these responses are mediated may differ between the 2 diseases.

The PI3K-Akt signaling pathway was mutually down-regulated in the tuberculosis-DM, tuberculosis-IH, and melioidosis with DM patient groups compared with their respective healthy controls. This signaling pathway regulates many cellular functions, such as glucose metabolism, apoptosis, and immune response [38], and PI3K-Akt dysfunction impairs phagocyte function in sepsis [39]. Furthermore, PI3K signaling influences T-cell differentiation [40]. Collectively, these findings suggest that the PI3K-Akt pathway is a crucial functional capacity of immune cells. Down-regulation of this pathway, as observed in the patients with DM, may lead to impaired innate immune responses, including defective phagocytosis and killing mechanisms, and a reduced capacity for control of intracellular infection. Several bioactive compounds, such as kaempferol, quercetin, and berbamine, have been shown to restore Akt phosphorylation and downstream glucose transporter 4 translocation in insulin-resistant cells via the insulin receptor substrate 1/PI3K/Akt axis, directly addressing the pathway suppression observed in the current study [41]. Of these, berbamine has the strongest anti-infective rationale, clearing both drug-sensitive and drug-resistant Mtb in macrophages through ROS/calcium-dependent autophagy activation, while simultaneously improving insulin resistance [42]. Our findings therefore support further exploration of the impact of these host-directed therapies in DM.

In the current study, we reanalyzed data from Eckold et al [25], and we identified further genes potentially playing deleterious roles in patients with tuberculosis and DM. Two hub genes, *CEACAM8* and *BPI*, were differentially expressed in the tuberculosis-DM compared with the tuberculosis-only patient groups, and recent evidence provides important mechanistic context for interpreting their up-regulation in this comorbid condition. *CEACAM8* encodes a cell adhesion module (CD66b), a glycosylphosphatidylinositol-anchored glycoprotein stored in the gelatinase granules of neutrophils and rapidly mobilized to the plasma membrane on activation. Unlike *CEACAM1* and *CEACAM3, CEACAM8* does not directly bind known intracellular pathogens; instead, surface up-regulation and shedding of a soluble form function as downstream readouts of granule exocytosis and systemic neutrophil activation [43]. CD66b is highly expressed on neutrophils during activation [44] and is linked to increased disease severity and poor outcome in sepsis [45]. The elevated CEACAM8 observed in tuberculosis-DM may actively impair phagosomal killing of Mtb by arresting phagolysosome maturation.

This is supported by studies in type 2 DM (T2DM) demonstrating constitutive neutrophil activation, impaired glucose metabolism, and markedly reduced phagosome maturation and intracellular bacterial killing in high-glucose conditions [46, 47]. Hyperglycemia-driven NF-κB activation and diacylglycerol-mediated reduced (hydrogenated) nicotinamide adenine dinucleotide phosphate (NADPH) oxidase assembly sustain this feed-forward loop, producing chronically activated but functionally exhausted neutrophils with up-regulated surface CD66b [46]. *BPI* encodes bactericidal/permeability-increasing (BPI) protein, which is found in the azurophilic granules of neutrophils and has binding specificity and neutralization activity against lipopolysaccharide from gram-negative bacteria [48]. In tuberculosis, BPI protein can recognize lipoarabinomannan of Mtb which shares some similar properties with lipopolysaccharide; BPI protein–mediated immune response to Mtb can thus be detectable in patients with tuberculosis [49].

A study in recent years demonstrated that recombinant BPI protein is rapidly internalized into Mtb-infected macrophages and significantly reduces intracellular mycobacterial growth. However, BPI protein simultaneously suppressed TNF production [50]. Given that TNF is indispensable for controlling Mtb infection [51], BPI protein–mediated TNF suppression presents a mechanistic paradox: while BPI protein exerts direct bactericidal activity, its concurrent dampening of the TNF axis may compromise the macrophage-mediated adaptive immune response needed for durable bacterial containment. The intersection of CEACAM8 and BPI protein dysregulation is mechanistically relevant. Rather than reflecting effective antimicrobial deployment, this sustained granule release is more consistent with a state of neutrophil exhaustion contributing to tuberculosis-DM immunopathology. Future work should directly test whether pharmacological restoration of phagosomal function or selective BPI protein augmentation without concurrent TNF suppression can rescue intracellular mycobacterial killing in neutrophils and macrophages in patients with tuberculosis-DM. Agents such as chlorine-amidine reduces neutrophil extracellular trap formation and inflammatory burden in diabetic murine models without abolishing other neutrophil bactericidal functions, including phagocytosis and ROS generation [52].

Cell-type deconvolution analysis identified enriched class-switched memory B cells and intermediate hyperglycemia cells in patients with melioidosis and T2DM compared with the non-DM group. Enriched class-switched memory B cells in patients with melioidosis and T2DM suggests the presence of increased Bp-specific memory B cells capable of generating faster and more effective immune response against Bp infection. Increased Bp-specific antibody levels in patients with melioidosis with T2DM was associated with survival, implying alternative immune responses against melioidosis with T2DM [53]. intermediate hyperglycemia cells are an important cellular source of IFN-γ in early host response against Bp infection [54]. Morris et al [55] demonstrated delayed IFN-γ response from IFN-γ–producing cells (NK, intermediate hyperglycemia, and T cells) against Bp infection in T2DM mice compared with nondiabetic mice. However, these cells were later increased in T2DM mice concomitantly with heightened proinflammatory cytokines and increased bacterial burden.

While DM increases susceptibility to melioidosis much more than susceptibility to tuberculosis (12-fold vs 3-fold), our study found a greater impact of DM on the transcriptome in tuberculosis. This may be due to differences in the pathogenesis of Bp and Mtb infection. In addition, detection of DM-related transcriptomic changes in melioidosis may be obscured by the “noise” of the heightened inflammatory response associated with acute sepsis, wherein neutrophil-driven innate immune activation exerts a dominant effect [56]. Melioidosis frequently presents as fulminant systemic infection, in which acute inflammation overwhelmingly shapes the host transcriptional landscape. More broadly, sepsis is characterized by the coexistence of hyperinflammation and immune suppression, leading to substantial transcriptional variability and interindividual heterogeneity such that the underlying biological processes of Bp sepsis are likely to mask more subtle host modifiers such as DM [57]. In contrast, the less acute nature of tuberculosis with pulmonary focus enabled easier discernment of DM-related changes.

There were some limitations in the current study. The melioidosis cohort was recruited from a single-center study that may not be globally representative of patients with melioidosis. The study site is a tertiary hospital, where patients with melioidosis were transferred from adjacent provinces, resulting in delayed enrollment, diagnosis, and treatment. Due to a lack of information on interventions or treatments received before enrollment into the studies, the impact of drugs on the observed transcriptomic profiles is unknown. This is particularly relevant for antidiabetic therapies such as metformin, which have well-described immunomodulatory effects. Experimental and clinical studies have shown that metformin can alter host responses during intracellular infections, including enhancement of macrophage antimicrobial functions, modulation of cytokine production, and regulation of cellular metabolism via adenosine monophosphate–activated protein kinase–dependent pathways [58]. In the context of Mtb, metformin has been reported to enhance phagocytosis and ROS production in human peripheral blood mononuclear cells, while also dampening excessive inflammatory responses [58].

Similar host-directed effects have been observed in diabetic mouse models, in which metformin improved infection outcomes and reduced immunopathology [59]. Therefore, prior metformin use may have attenuated or reshaped the observed hyperinflammatory transcriptomic signatures in the DM cohorts, suggesting that our findings may be conservative Participants were enrolled by the time of established disease, with a median of 5 days after hospital admission due to the use of Bp-positive culture as an inclusion criterion. Therefore, early transcriptomic responses adjacent to disease onset were not captured. Most patients with melioidosis had ≥1 comorbid condition, which may introduce confounding factors in delineating the transcriptomic profile associated with DM. Accurate disease severity scoring was unavailable, which may have limited our ability to fully detect the impact of DM in melioidosis. Smoking status, which can affect host inflammatory responses [60], was also unavailable for our cohorts.

Finally, full characterization of patients’ DM status, including type (type 1, type II, or other), length of disease, and treatment, was not available in this study, and these features may have influenced outcomes. Furthermore, the tuberculosis cohorts originated from different geographic regions, which may introduce variability in host genetics, environmental exposures, and pathogen diversity, potentially influencing transcriptomic profiles and confounding direct comparisons between diseases. However, despite geographic heterogeneity, consistent pathway-level findings across tuberculosis cohorts support the robustness of our findings.

**Figure 5.**
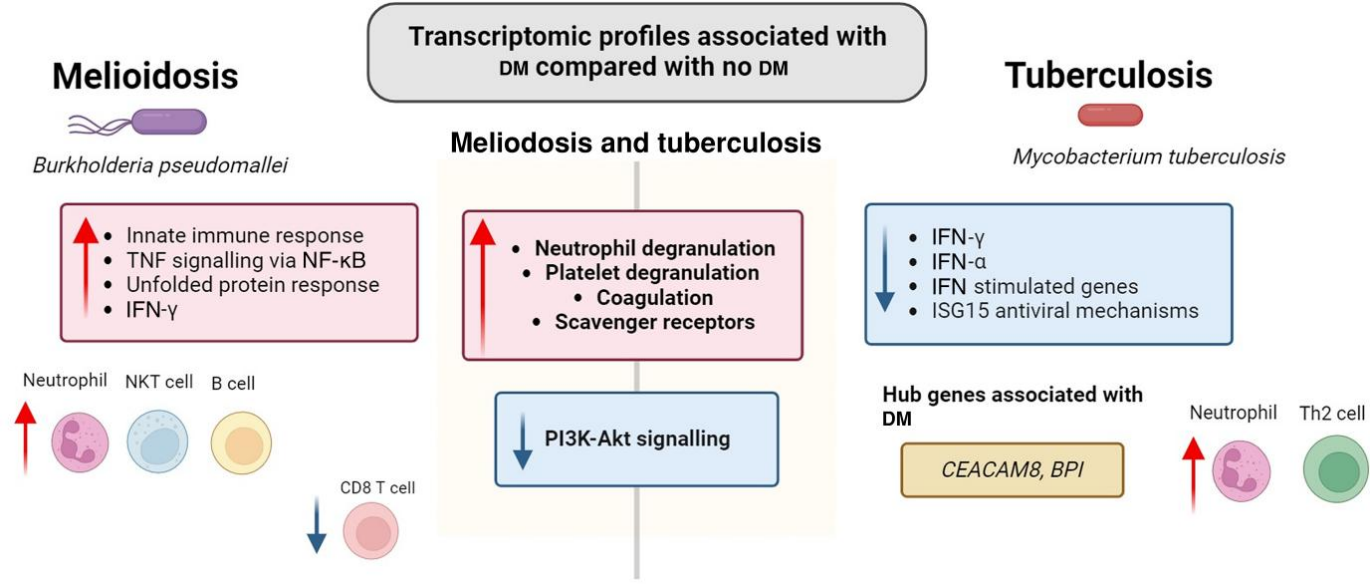
Graphic summary of whole-blood transcriptomic profiles associated with melioidosis and tuberculosis in people who live with or without diabetes mellitus (DM). compared with their respective healthy control cohorts (figure created with BioRender.com). Abbreviations: IFN, interferon; ISG15, IFN-stimulated gene 15; intermediate hyperglycemia, natural killer T; PI3K-Akt, phosphoinositide 3-kinase protein kinase B; Th2, T-helper 2; TNF, tumor necrosis factor.

In summary, both patients with melioidosis and those with tuberculosis with DM exhibit increased inflammatory responses compared with patient without DM, with key dysfunctions of neutrophil function, clotting cascades, and platelet function (Figure 5). The reduced IFN immune response in patients with tuberculosis with DM may be a potential underlying mechanism of increased susceptibility to Mtb infection. Unlike tuberculosis, melioidosis is typically an acute infection, characterized by more pronounced activation of neutrophil degranulation, coagulation, and platelet activation pathways. Animal models are needed to determine whether the increased neutrophil-mediated inflammation is a compensatory response to uncontrolled infection. Alternatively, models must assess whether this inflammation causes impaired cellular IFN and T-cell responses through immunomodulation and innate cell dysregulation. Future use of a diabetic mouse model along with studying early transcriptomic responses to vaccines in people with or without DM will build on our findings. These findings, across 2 diseases and 5 countries, give a general insight into the impact of DM on the human immune response to infection, but studies in further diseases and populations will extend the generalizability of our findings.

This study provides new evidence that DM is associated with a shared, dysregulated host immune response across 2 major intracellular infections. Clinically, these findings suggest that patients with DM represent a distinct immunological subgroup in whom conventional antimicrobial therapy alone may be suboptimal. The identification of impaired PI3K-Akt signaling and exaggerated neutrophil-driven inflammation highlights potential targets for host-directed therapy. Future work should prioritize validation of these pathways in independent cohorts and experimental models, alongside evaluation of targeted interventions aimed at restoring immune balance to improve outcomes in this high-risk group.

## Supplementary Material

ofag286_Supplementary_Data

## Supplementary Data

Supplementary materials are available at *Open Forum Infectious Diseases* online. Consisting of data provided by the authors to benefit the reader, the posted materials are not copyedited and are the sole responsibility of the authors, so questions or comments should be addressed to the corresponding author.
