# Supplementary material for: Association of Diabetes Mellitus With a Shared Hyperinflammatory Immune Response in Patients With Melioidosis and Patients With Tuberculosis: An Observational Case-Control Study
Source: Open Forum Infect Dis. 2026 Jun 17;13(6):ofag286. doi: 10.1093/ofid/ofag286 (PMC13274303; doi:10.1093/ofid/ofag286)
Supplement: ofag286_Supplementary_Data [file ofag286_supplementary_data.zip › Re_Supp info_T2DM during melio and TB_OFID_10.4.26_clean.docx]

**Supplementary information**

**Supplementary Table 1.** Validation of differentially expressed genes derived from melioidosis patients with diabetes compared to healthy donors by measuring serum proteins from the same cohort using a multiplex cytokine array on the Mesoscale Discovery platform.

| **Target proteins** | **Differentially expressed genes** | **Log2FoldChange** | **Adjust p-value** |
| --- | --- | --- | --- |
| IL-27 | *IL27* | 2.318505065 | 3.36E-05 |
| ICAM-1 | *ICAM1* | 2.052747213 | 5.67E-09 |
| IL-1β | *IL1B* | 1.496463191 | 0.000181734 |
| IL-10 | *IL10* | 1.220154645 | 0.029564745 |
| IL-15 | *IL15* | 0.894454411 | 0.008173798 |
| TNF-α | *TNF* | 0.642364787 | 0.04222255 |
| PlGF | *PIGF* | 0.376901225 | 0.00082796 |
| IL-16 | *IL16* | -0.342375959 | 0.045563584 |
| IL-7 | *IL7* | -0.664295413 | 0.0069012 |
| IL-23 | *IL23A* | -1.282588587 | 0.001259597 |
| IL-4 | *IL4* | -1.309451141 | 0.028632183 |
| IL-2 | *IL2* | -2.17917031 | 0.032326781 |
| MCP-1 | *CCL2* | -2.378923019 | 0.000177369 |

**Supplementary Figure 1. Melioidosis drives widespread immune activation.** (A) Volcano plot of differentially expressed genes between uninfected diabetes (DM) outpatients and healthy donors. (B) Volcano plot of differentially expressed genes between melioidosis patients and uninfected control cohort. Differentially expressed genes were based on absolute (Log_2_ fold-change)$\geq$ 1 (x-axis) and adjusted P-value < 0.05 (y-axis) (dotted lines). (C) Functional pathway analysis based on Reactome gene sets following differential gene expression (DE) analysis between melioidosis patients and uninfected healthy donors. The gradient colour bar corresponds to the adjusted P-value. The size of each term is indicated by representative counts (number of DEGs). Differentially expressed genes were pre-filtered based on a cut-off of absolute[Log2 fold-change$]\geq$1 and adjusted P-value <0.05. MelvsHctrl_up and MelvsHctrl_down = up and downregulated pathways derived from DE analysis between melioidosis patients compared to uninfected healthy donors respectively.


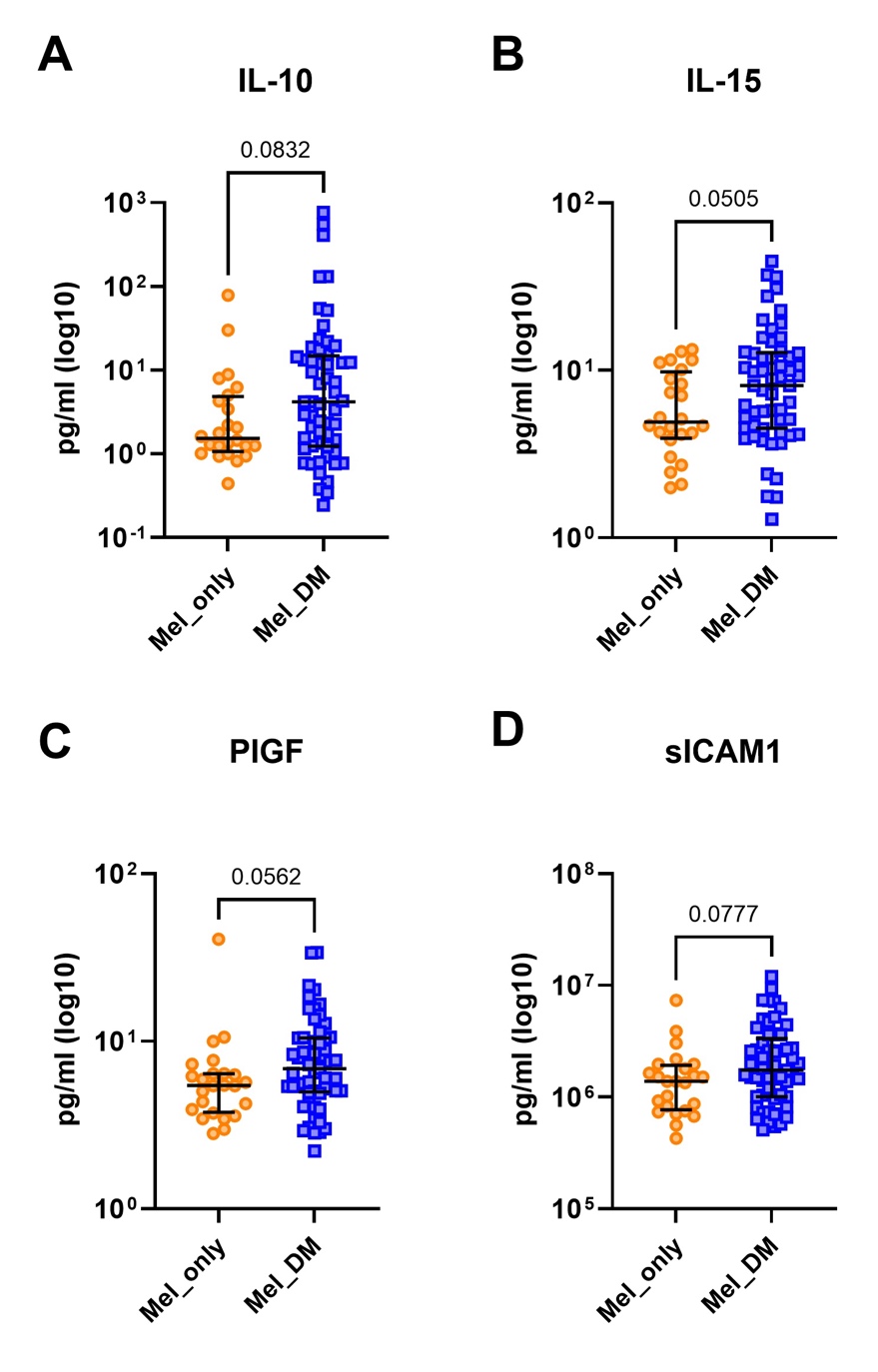


**Supplementary Figure 2**. **A trend of increased inflammatory responses in plasma from melioidosis patients with diabetes.** (A) IL-10, (B) IL-15, (C) placental growth factor (PIGF) and (D) soluble intercellular adhesion molecule 1 (sICAM1) were measured in serum of acute melioidosis patients with (N=56, Mel_DM) and without DM (n=24, Mel_only) using a multiplex cytokine array on the Mesoscale Discovery platform. Data are presented as scatter plots with median and interquartile range. Statistical differences were tested by Mann-Whitney test and p<0.05 is considered statistically significant.

**Supplementary Figure 3. Higher magnitude of transcriptomic response in melioidosis patients with DM.** (A) Volcano plot of differentially expressed genes between melioidosis patients without diabetes and healthy donors. (B) Volcano plot of differentially expressed genes between melioidosis patients with DM and healthy donors. Differentially expressed genes (DEGs) were based on absolute (Log_2_ fold-change)$\geq$ 1 (x-axis) and adjusted P-value < 0.05 (y-axis) (dotted lines). (C) Euler diagram shows overlapped and distinct DEGs derived from DE analyses among melioidosis patients and healthy donors (A, B). Up-DEGs in Melioidosis-only (magenta) and Down-DEGs in Melioidosis-only (blue) = up and downregulated genes derived from DE analysis between melioidosis patients without diabetes and healthy donors. Up-DEGs in Melioidosis-DM (orange) and Down-DEGs in Melioidosis-DM (green) = up and downregulated genes derived from DE analysis between melioidosis patients with diabetes and healthy donors.

**Supplementary Figure 4. Higher magnitude of transcriptomic responses in tuberculosis patients with intermediate hyperglycaemia and diabetes from South African cohort.** (A) Principal component analysis (PCA) of the top 1,000 most variable genes among TB patients and control cohorts. TB cohort is divided into the patients with diabetes (“dm”, orange dots, n=19), without diabetes (“nondm”, green dots, n=11), without intermediate hyperglycaemia (“inter”, green dots, n=15), uninfected diabetes outpatients (“dm_ctrl”, grey dots, n=33), and uninfected healthy donors (“healthy_ctrl”, grey dots, n=24)**.** (B) Volcano plot of differentially expressed genes between tuberculosis (TB) patients without diabetes (TB-only) and healthy donors. (C) Volcano plot of differentially expressed genes between TB patients with intermediate hyperglycaemia (TB-IH) and healthy donors. (D) Volcano plot of differentially expressed genes between TB patients with diabetes (TB-DM) and healthy donors. Differentially expressed genes (DEGs) were based on absolute (Log_2_ fold-change)$\geq$ 1 (x-axis) and adjusted P-value < 0.05 (y-axis) (dotted lines). (E) Euler diagram shows overlapped and distinct DEGs derived from DE analyses among TB patients and healthy donors (B-D). Up-DEGs in TB-only (magenta) and Down-DEGs in TB-only (blue) = up and downregulated genes derived from DE analysis of TB patients without DM compared to healthy donors. Up-DEGs in TB-IH (red) and Down-DEGs in TB-IH (grey) = up and downregulated genes derived from DE analysis of TB patients with intermediate hyperglycaemia compared to healthy donors. Up-DEGs in TB-DM (orange) and Down-DEGs in TB-DM (green) = up and downregulated genes derived from DE analysis of TB patients with DM compared to healthy donors.

**Supplementary Figure 5. Increased inflammatory immune responses in melioidosis and tuberculosis patients with diabetes.** Functional pathway analysis based on KEGG gene sets following differential gene expression (DE) analysis between melioidosis patients compared to uninfected healthy donors and tuberculosis (TB) patients compared to uninfected healthy donors. (A) Up-regulated KEGG pathways derived from the DE analyses among TB and melioidosis patients compared to their respective uninfected healthy control cohorts. (B) Down-regulated KEGG pathways derived from the DE analyses among TB and melioidosis patients compared to their respective uninfected healthy control cohorts. The gradient colour bar corresponds to the adjusted P-value. The size of each term is indicated by representative counts (number of DEGs). Differentially expressed genes were pre-filtered based on a cut-off of absolute[Log2 fold-change$]\geq$1 and adjusted P-value <0.05. Gene set enrichment analysis based on Hallmark gene sets following differential gene expression (DE) analysis among tuberculosis patients across four study sites. (C) TB patients with intermediate hyperglycaemia (TB-IH, n=44) compared to TB patients without DM (TB-only, n=46). (D) TB patients with diabetes (TB-DM, n=61) compared to TB patients without DM (TB-only, n=46). Normalised enrichment scores are displayed, in which pathways were deemed significant when adjusted P-value <0.05.

**Supplementary Figure 6. Higher magnitude of transcriptomic responses in tuberculosis patients with intermediate hyperglycaemia and diabetes from Romania cohort.** (A) Principal component analysis (PCA) of the top 1,000 most variable genes among TB patients and control cohorts. TB cohort is divided into the patients with diabetes (“dm”, orange dots, n=15), without diabetes (“nondm”, green dots, n=10), without intermediate hyperglycaemia (“inter”, green dots, n=10), uninfected diabetes outpatients (“dm_ctrl”, grey dots, n=19), and uninfected healthy donors (“healthy_ctrl”, grey dots, n=12)**.** (B) Volcano plot of differentially expressed genes between tuberculosis (TB) patients without diabetes (TB-only) and healthy donors. (C) Volcano plot of differentially expressed genes between TB patients with intermediate hyperglycaemia (TB-IH) and healthy donors. (D) Volcano plot of differentially expressed genes between TB patients with diabetes (TB-DM) and healthy donors. Differentially expressed genes (DEGs) were based on absolute (Log_2_ fold-change)$\geq$ 1 (x-axis) and adjusted P-value < 0.05 (y-axis) (dotted lines). (E) Euler diagram shows overlapped and distinct DEGs derived from DE analyses among TB patients and healthy donors (B-D). Up-DEGs in TB-only (magenta) and Down-DEGs in TB-only (blue) = up and downregulated genes derived from DE analysis of TB patients without DM compared to healthy donors. Up-DEGs in TB-IH (red) and Down-DEGs in TB-IH (grey) = up and downregulated genes derived from DE analysis of TB patients with intermediate hyperglycaemia compared to healthy donors. Up-DEGs in TB-DM (orange) and Down-DEGs in TB-DM (green) = up and downregulated genes derived from DE analysis of TB patients with DM compared to healthy donors. (F) Euler diagram shows overlapped and distinct DEGs derived from DE analyses between South African cohort and Romania cohort. South African cohort: Up-DEGs in TB-DM (magenta) and Down-DEGs in TB-DM (blue) = up and downregulated genes derived from DE analysis of TB patients with DM compared to healthy donors. Romania cohort: Up-DEGs in TB-DM (red) and Down-DEGs in TB-DM (green) = up and downregulated genes derived from DE analysis of TB patients with DM compared to healthy donors.

**Supplementary Figure 7. Increased inflammatory immune responses in tuberculosis patients with diabetes and intermediate hyperglycaemia from Romania cohort.** Functional pathway analysis based on Reactome gene sets following differential gene expression (DE) analysis between melioidosis patients compared to uninfected healthy donors and tuberculosis (TB) patients compared to uninfected healthy donors. (A) Up- and down-regulated Reactome pathways derived from the DE analyses among TB patients compared to their respective uninfected healthy control cohorts. The gradient colour bar corresponds to the adjusted P-value. The size of each term is indicated by representative counts (number of DEGs). Differentially expressed genes were pre-filtered based on a cut-off of absolute [Log2 fold-change$]\geq$1 and adjusted P-value <0.05. (B) Gene set enrichment analysis based on Hallmark gene sets following differential gene expression (DE) analysis among tuberculosis patients from Romania cohort. (C) TB patients with intermediate hyperglycaemia (TB-IH, n=10) compared to TB patients without DM (TB-only, n=10). (D) TB patients with diabetes (TB-DM, n=15) compared to TB patients without DM (TB-only, n=10). Normalised enrichment scores are displayed, in which pathways were deemed significant when adjusted P-value <0.05.

**Supplementary Figure 8. Reduced pro-inflammatory responses and cellular signalling in melioidosis and TB with diabetes compared to uninfected diabetes control.** (A) Volcano plot of differentially expressed genes between melioidosis patients with diabetes and uninfected diabetes control. Differentially expressed genes (DEGs) were based on absolute (Log_2_ fold-change)$\geq$ 1 (x-axis) and adjusted P-value < 0.05 (y-axis) (in coloured dots). (B) Euler diagram shows overlapped and distinct DEGs derived from DE analyses among melioidosis patients with diabetes compared to healthy donors or diabetes control. Up-DEGs in Melioidosis-DM (magenta) and Down-DEGs in Melioidosis-DM (blue) = up and downregulated genes derived from DE analysis of melioidosis with diabetes compared to diabetes control. Up-DEGs in Melioidosis-DM (orange) and Down-DEGs in Melioidosis-DM (green) = up and up and downregulated genes derived from DE analysis of melioidosis patients with diabetes compared to healthy donors. (C) Volcano plot of differentially expressed genes between TB patients with diabetes and uninfected diabetes control from South African cohort. (D) Euler diagram shows overlapped and distinct DEGs derived from DE analyses among TB patients with diabetes compared to healthy donors or diabetes control. Up-DEGs in TB-DM (magenta) and Down-DEGs in TB-DM (blue) = up and downregulated genes derived from DE analysis of TB patients with diabetes compared to diabetes control. Up-DEGs in TB-DM (orange) and Down-DEGs in TB-DM (green) = up and up and downregulated genes derived from DE analysis of TB patients with diabetes compared to healthy donors. (E-F) Up- and down-regulated Reactome pathways derived from the DE analyses among melioidosis and TB patients compared to their respective uninfected diabetes control cohorts. The gradient colour bar corresponds to the adjusted P-value. The size of each term is indicated by representative counts (number of DEGs). Differentially expressed genes were pre-filtered based on a cut-off of absolute [Log2 fold-change$]\geq$1 and adjusted P-value <0.05.

**Supplementary Figure 9. Increased inflammatory immune responses in tuberculosis patients with diabetes.**

Weighted gene co-expression network analysis (WGCNA) was performed in 107 tuberculosis (TB) patients including 61 TB patients with diabetes (DM) and 46 TB patients without diabetes. (A) Sample dendrogram identified and removed one outlier. (B) Sample dendrogram with corresponding clinical data after removing the outlier. (C) Scale-free topological analysis, with scale independence plot indicates scale free topology model fit (R^2^) and mean connectivity plot with soft threshold on x-axis. (D) Cluster dendrogram of co-expressed module (module eigengene, ME).

**Supplementary Figure 10**. **Enriched neutrophils and T helper 2 cells were associated with diabetes during tuberculosis.** (A, B) Enrichment of neutrophils and T helper 2 (Th2) cells between tuberculosis patients with diabetes and without diabetes respectively. Cell enrichment was performed using xCell deconvolution method. The statistical analysis was performed using Mann-Whitney test, and the corresponding P-value was displayed on each plot along with median and inter-quartile range boxes.

**Supplementary materials and methods**

1. **Measurement of serum proteins from acute melioidosis**

Measurement of cytokines in serum was performed using the Mesoscale discovery (MSD) V-PLEX Human Biomarker 54-Plex Kit according to manufacturer's instructions. Briefly, each plate was pre-coated with capture monoclonal antibodies on pre-defined spots on each well. The plates were washed using a wash buffer prior to addition of 50µl diluted serum samples or calibrator or controls per well and was incubated for 2 hours at room temperature while shaking on a plate shaker at 1000rpm. Calibrators were 4-fold diluted with respective diluents and run in duplicates. Clinical samples were run in singlets. The plates were then washed with wash buffer three times before the addition of 25µl of analyte specific detection antibody and left to incubate for 2 hours while shaking. Lastly, plates were washed three times with wash buffer and 150µl of Read Buffer T was added prior to analysis of the plate immediately on the MSD Sector QuickPlex SQ 120MM Reader. Data analysis was performed using the MSD Workbench 4.0 software, which generates a standard curve from the calibrators to determine the concentration of the inflammatory marker.

1. **Core upstream data analysis**

For melioidosis cohort FASTQ files were retrieved from the FTP server provided by the OGC, WCHG sequencing provider. Upstream data processing, including read alignment and quantification, was carried out on the computing cluster maintained by the Computational Biology Research Group at the Weatherall Institute of Molecular Medicine, University of Oxford. For tuberculosis cohort, *Eckold et al (1)* generated the expression data and provided the raw sequencing data (FASTQ files). Data from both cohorts were processed using the same pipeline.

Initial quality control of raw sequencing data was performed using FastQC (v0.11.9). Reads were then aligned to the human reference genome (UCSC hg38.p2, version 20201) using STAR aligner (v2.6.1d), generating 440 BAM files. These aligned reads were visualized with the Integrative Genomics Viewer (IGV, v2.4.16). Read quantification was performed using the featureCounts function from the Subread package (v1.6.2), counting uniquely mapped reads. The resulting tab-delimited count files were compiled into a single expression matrix using the Sartools R package (v1.6.8), which served as the input for downstream analyses.

Reference:

1. C. Eckold *et al.*, Impact of Intermediate Hyperglycemia and Diabetes on Immune Dysfunction in Tuberculosis. *Clin Infect Dis* **72**, 69-78 (2021).
