# Supplementary figures and images for: Association of Diabetes Mellitus With a Shared Hyperinflammatory Immune Response in Patients With Melioidosis and Patients With Tuberculosis: An Observational Case-Control Study
Source: Open Forum Infect Dis. 2026 Jun 17;13(6):ofag286. doi: 10.1093/ofid/ofag286 (PMC13274303; doi:10.1093/ofid/ofag286)

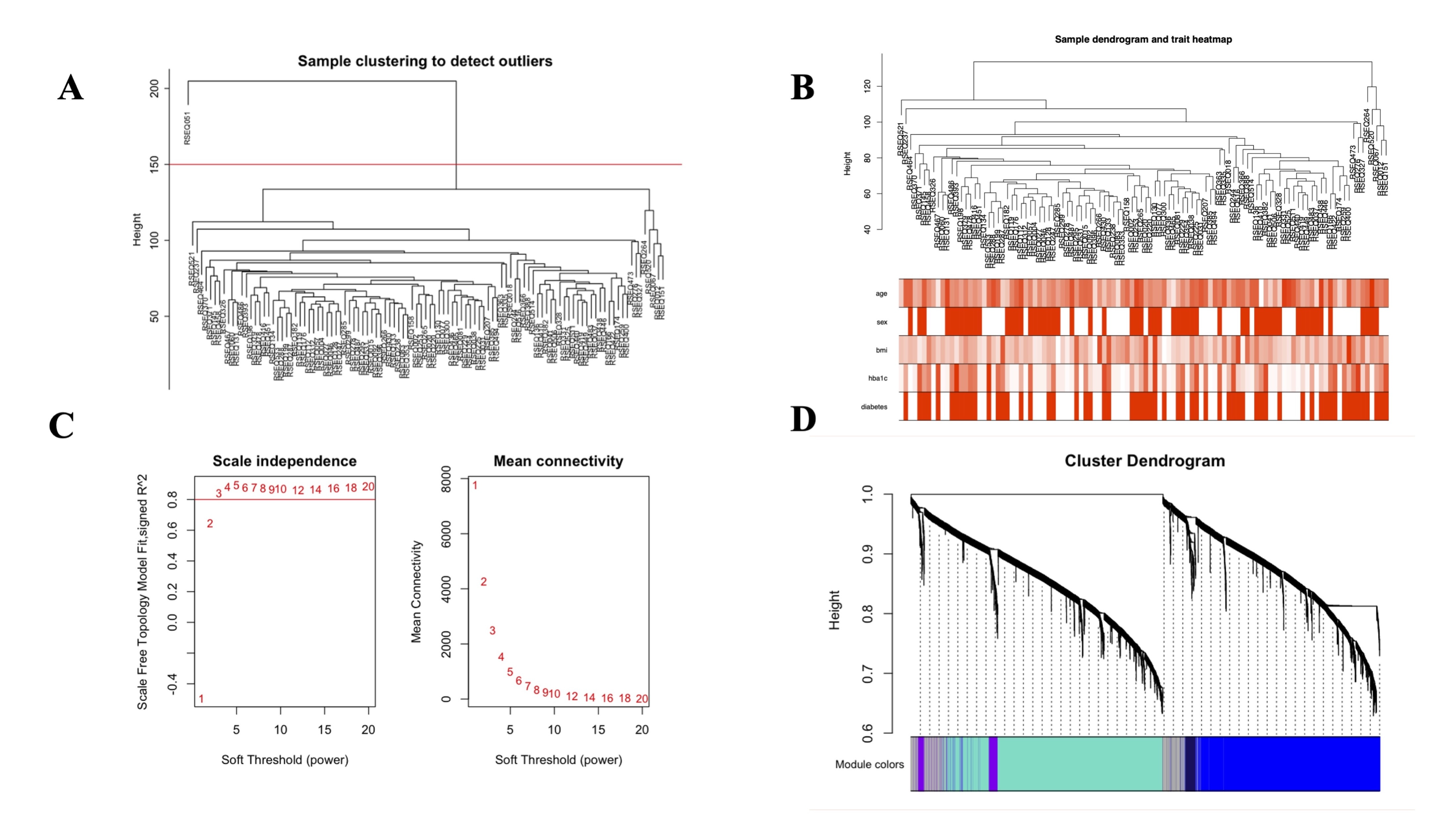

Supplement: ofag286_Supplementary_Data [file ofag286_supplementary_data.zip › Figure S9.tiff]

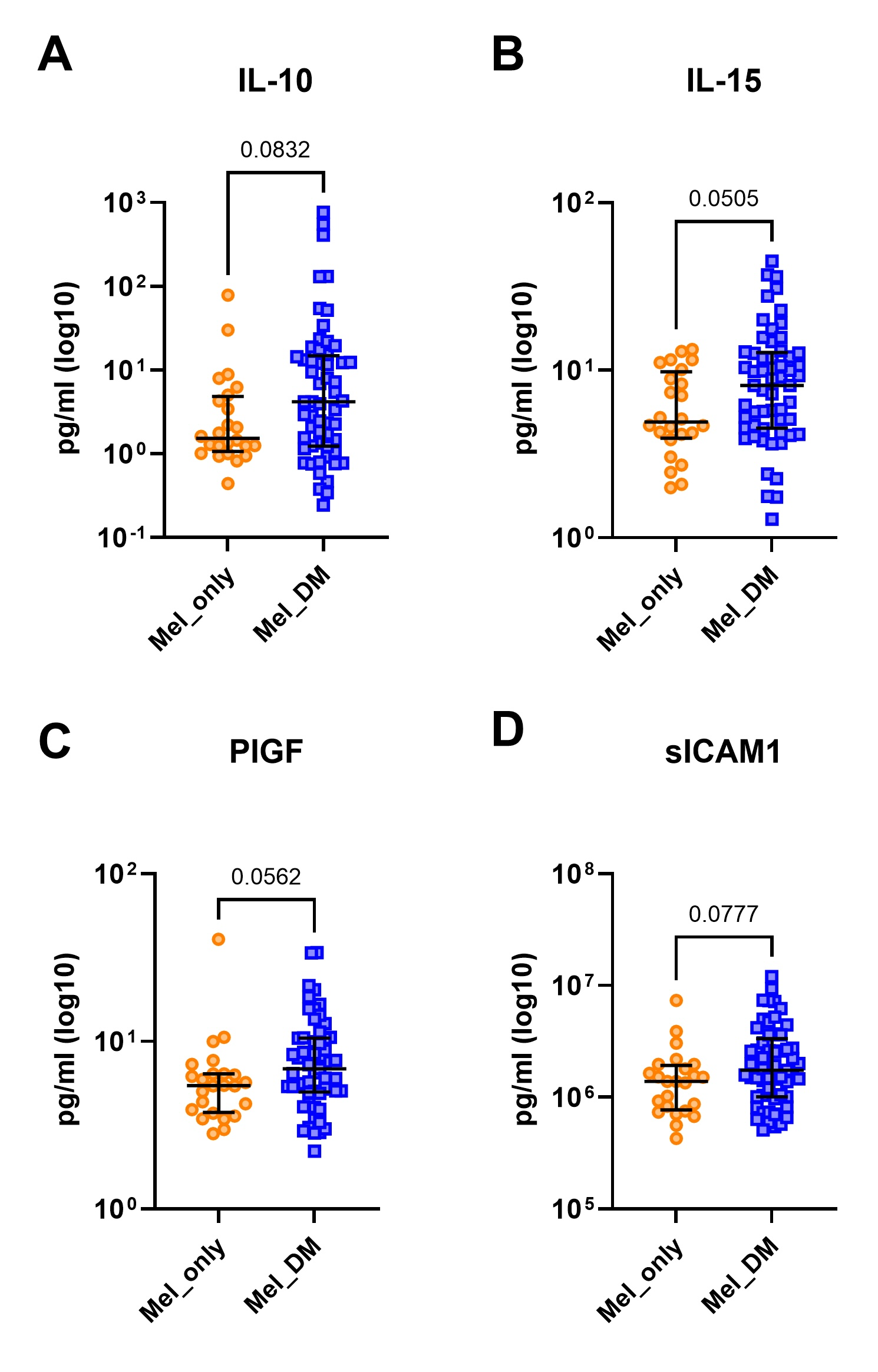

Supplement: ofag286_Supplementary_Data [file ofag286_supplementary_data.zip › Figure S2.tiff]

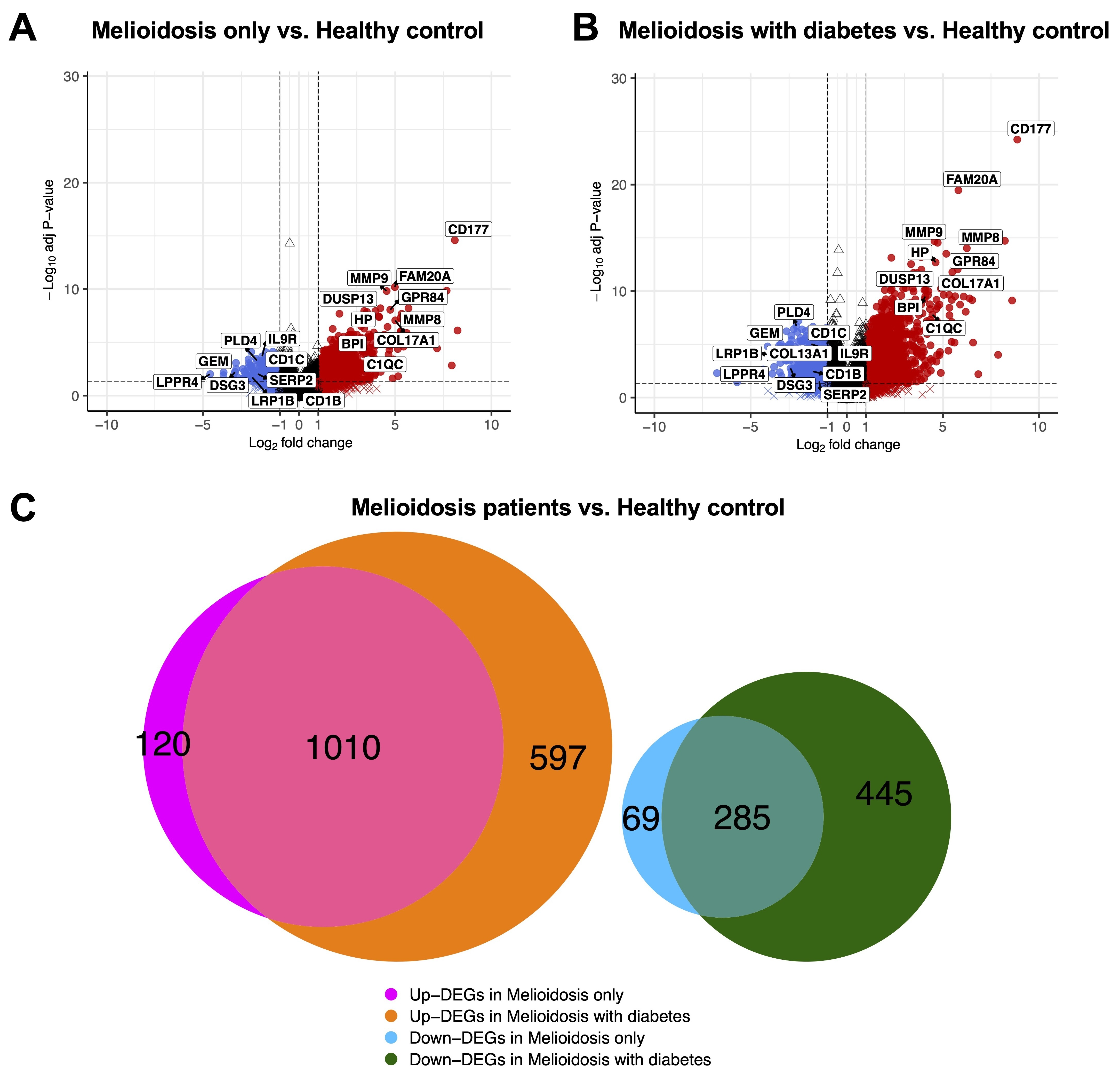

Supplement: ofag286_Supplementary_Data [file ofag286_supplementary_data.zip › Figure S3.tiff]
